# Long-Term Epidemiology of Hepatitis B and Impact of Vaccination in the Autonomous Province of Vojvodina, Serbia: A Population-Based Study

**DOI:** 10.3390/microorganisms13112504

**Published:** 2025-10-31

**Authors:** Tatjana Pustahija, Snežana Medić, Mioljub Ristić, Zagorka Lozanov Crvenković, Smiljana Rajčević, Svetlana Ilić, Teodora Turudija, Stevan Stojanović, Dušan Petrović, Vladimir Petrović

**Affiliations:** 1 Faculty of Medicine, University of Novi Sad, 21000 Novi Sad, Serbia; snezana.medic@mf.uns.ac.rs (S.M.); mioljub.ristic@mf.uns.ac.rs (M.R.); smiljana.rajcevic@mf.uns.ac.rs (S.R.); stevan.stojanovic@mf.uns.ac.rs (S.S.); vladimir.petrovic@mf.uns.ac.rs (V.P.); 2 Institute of Public Health of Vojvodina, 21000 Novi Sad, Serbia; svetlana.ilic@izjzv.org.rs (S.I.); dusan.petrovic@izjzv.org.rs (D.P.); 3 Faculty of Science, University of Novi Sad, 21000 Novi Sad, Serbia; zlc@dmi.uns.ac.rs; 4 Health Center Kikinda, 23300 Kikinda, Serbia; teodora.turudija@gmail.com; 5 Clinic of Urology, University Clinical Center of Vojvodina, 21000 Novi Sad, Serbia

**Keywords:** hepatitis B, epidemiology, trends, prevention, vaccine coverage, vaccination impact

## Abstract

This retrospective population-based study aimed to update the epidemiology of hepatitis B in Vojvodina, Serbia, and assess the impact of vaccination on acute and chronic forms of the disease. Surveillance data from 1978 to 2024 were analyzed by period, age/sex, and geographic distribution. Joinpoint and Poisson regression analyses were used to evaluate long-term trends in hepatitis B incidence and mortality rates and the vaccine-coverage effects, respectively. A decreasing trend in both acute and chronic hepatitis B incidence and mortality was observed over the study period. The average incidence rate declined from 11.51/100,000 in the pre-immunization period (1978–1987) to 1.68/100,000 during the universal immunization period (2006–2024). For chronic hepatitis B, the average incidence rates were 2.27 and 2.62/100,000 in the periods 1997–2005 and 2006–2024, respectively. Uneven spatial distribution was noted across municipalities. Poisson regression analysis showed that for every 1% increase in infant vaccine coverage in the previous year, the incidence rates of acute and chronic hepatitis B decreased significantly by 2.20% and 1.80%, respectively. These findings support the sustained effectiveness of hepatitis B vaccination, particularly among children and adolescents, and underscore the importance of addressing subnational disparities.

## 1. Introduction

Hepatitis B virus (HBV) infection is associated with high global morbidity and mortality, including acute and chronic hepatitis, liver cirrhosis and hepatocellular carcinoma [1]. The World Health Organization (WHO) has created a strategy to reduce the incidence of HBV by 90%, mortality by 65% and HBsAg prevalence among children under five to less than 0.1%, by 2030 [2,3]. Although HBV may be prevented by immunization, it remains a global health concern. According to WHO estimates, 254 million people lived with chronic HBV in 2022 (3.2% of the world population), leading to 1.1 million deaths, primarily due to cirrhosis and hepatocellular carcinoma [4,5]. In the same year, 30 European Union/European Economic Area (EU/EEA) member states reported 28,855 HBV cases, corresponding to a notification rate of 8.5 cases per 100,000 population. The highest percentage of HBV reported cases was chronic forms of the disease (40%), while acute forms represented 7% of cases. Nearly half (47%) of the cases were classified as unknown [6,7].

Serbia has a population of approximately 6.7 million. Of these, about 1.7 million people (25% of the population) reside in the Autonomous Province of Vojvodina (Vojvodina), situated in the north of the country [8]. Mandatory surveillance of acute HBV has been conducted in Serbia since 1978, and for chronic HBV since 1997 [9,10].

Vaccination against HBV contributed to the decreasing incidence of HBV worldwide, especially in children. In addition, other primary prevention measures have supported the reduction of HBV burden in the population [11,12]. In Serbia, mandatory immunization of newborns of HBsAg positive mothers and high-risk groups for HBV was introduced in 1988 [13,14]. Mandatory universal immunization of newborns and infants, along with catch-up immunization of children at the age of 12, using a three-dose schedule at 0, 1, and 6 months, began in 2005–2006 [13,14].

Since vaccination against HBV at the age of 12 was completed in 2018, vaccination is currently offered to children up to 18 years who missed earlier doses. Vaccination against HBV is also mandatory for high-risk groups. These include healthcare workers, sexual partners and household contacts of HBsAg-positive persons, intravenous drug users, prisoners and institutionalized people with developmental disabilities. Other covered groups are hemodialysis patients, insulin-dependent diabetics, patients with hemophilia and other diseases that require the use of blood derivatives, HIV and hepatitis C virus positive individuals, people with chronic liver and kidney diseases, recipients of liver or kidney transplants and patients with multiple sclerosis. Vaccination against HBV is recommended for people older than 18 years of age, who are negative for the presence of anti-HBs antibodies, i.e., those who were not vaccinated at birth or during elementary school [14,15,16].

In many European countries, a marked decline in HBV incidence and mortality has been observed following the introduction of universal immunization although the magnitude of this decline varies across countries [3]. However, long-term, population-based analyses evaluating both temporal trends in HBV incidence and mortality and the effects of vaccination remain scarce, particularly in Southeast Europe. To the best of our knowledge, no such studies have been conducted in Serbia at the national level. By using surveillance data from Vojvodina Province, this study provides novel insight in HBV epidemiology by analyzing incidence and mortality trends, their association with vaccination coverage in the preceding year, and disparities at the municipal level. A recent serosurvey conducted in Vojvodina [9] revealed 53.9% anti-HBs seropositivity of the population and a decreasing trend with age. This finding points to a substantial gap in knowledge regarding the 46.1% of individuals lacking immunity. Considering all the above, we aimed to quantify temporal changes in acute and chronic HBV incidence and mortality in Vojvodina (1978–2024) and to assess the association between vaccine coverage and disease reduction, with the ultimate goal of informing the national strategy for the elimination of this disease in Serbia by 2030.

## 2. Materials and Methods

In this retrospective population-level study, we determined trends in the incidence and mortality rates of acute HBV (1978–2024) and chronic HBV (1997–2024) registered in Vojvodina. In addition, we analyzed their topographical, age-specific, and gender-specific distributions. In addition, we analyzed the effects of vaccine coverage on acute and chronic HBV incidence rates. Data were obtained from the notifiable disease registry of communicable diseases maintained by the Institute for Public Health of Vojvodina (IPHV), which systematically collects reports of HBV cases from healthcare institutions and laboratories across the province in collaboration with six district IPHs. Completeness of reporting was not formally assessed, and no adjustments for potential underreporting were applied. Surveillance case definitions, aligned with WHO and European Centre for Disease Prevention and Control (ECDC) guidelines and updated over time, were applied. As diagnostic assays and reporting protocols evolved, case definitions were periodically updated in accordance with international recommendations. Consequently, due to these changes and incomplete laboratory confirmation in earlier decades, the definitions were not applied uniformly throughout the entire study period. Since 2017, surveillance has been based on the ECDC case definition. Accordingly, a laboratory-confirmed case of HBV required one or more of the following criteria: evidence of anti-HBc IgM antibodies and/or surface HBsAg and/or HBeAg and/or viral nucleic acid (HBV-DNA) in serum. In particular, acute HBV was defined by the detection of anti-HBc IgM antibodies and/or HBsAg or HBV-DNA in individuals with previous negative HBV markers within the past six months, while chronic HBV was defined by the presence of HBsAg, HBeAg, or HBV-DNA in the absence of anti-HBc IgM or their persistence for at least six months [17,18,19]. Data on immunization coverage against HBV were obtained from the annual reports of the IPHV on the implementation of the Program of mandatory immunizations in Vojvodina. The study timespan was divided into three distinct periods according to the implementation of HBV immunization in Serbia (Table 1).

Aggregated data on laboratory-confirmed cases of acute and chronic HBV were analyzed chronologically, demographically and topographically. The data on the mid-year number of inhabitants of Vojvodina, needed for the denominator in the calculation of incidence and mortality rates, were obtained from the website of the Republic Institute of Statistics of Serbia [8]. The annual crude incidence rate, expressed as the number of newly diagnosed cases of acute or chronic HBV per 100,000 population of Vojvodina, was calculated for each year of the study. Specific incidence rates were calculated by gender and by the following age groups: 0–9, 10–19, 20–29, 30–39, 40–49, 50–59 and ≥60 years. The incidence rate per 100,000 population was also calculated for each municipality in Vojvodina, as the ratio of the number of new cases of HBV to the number of inhabitants of the municipality. The mortality rate of acute and chronic HBV was calculated and expressed as the number of HBV-related deaths per 100,000 population. The case-fatality rate (CFR) from HBV was determined as the ratio of the number of HBV-related deaths to the number of all reported HBV cases and expressed as a percentage.

Changes in trends of HBV incidence and mortality rates of acute and chronic HBV for the study period, as well as for each age group separately, were assessed using joinpoint regression analysis, by estimating the annual percent change (APC) for each segment and the average annual percent change (AAPC), with corresponding 95% confidence interval (CI). If the AAPC is significantly different from zero at the alpha  =  0.05 level, we classified the trend (increasing or decreasing) as statistically significant. Joinpoint regression analysis allows detection of periods with distinct temporal patterns and quantifies the magnitude and direction of those changes. This approach is widely used in epidemiological trend analysis and is particularly suitable for our data, as the trend direction in incidence and mortality rates varied over the study period. We assessed the association between HBV immunization coverage in the previous year and acute and chronic HBV incidence rates between 1995 and 2024, using Poisson regression models. The HBV immunization coverage for the previous year (defined as the proportion of infants who completed the full three-dose series of the HBV vaccine) was used as a predictor variable, the number of reported HBV cases per year as the outcome variable and the population of Vojvodina as the exposure variable. The one-year lag was applied based on biological and epidemiological rationale, reflecting the expected time between immunization with three doses of the vaccine and measurable changes in HBV incidence. Model fit was evaluated by testing for overdispersion, which was not significant. The results are expressed as an incidence rate ratio (IRR) with 95% CI [20]. No additional covariates (e.g., age, sex, municipality) were included. Both the number of cases and vaccine coverage were treated as independent annual observations rather than time-series data. Autocorrelation was not formally tested, as the model was applied to aggregated annual data, which were considered independent. Stata version 16 (StataCorp LLC, College Station, TX, USA) and Joinpoint regression software version 5.0.2 (National Cancer Institute, Bethesda, MD, USA; available online: https://surveillance.cancer.gov/joinpoint/ (accessed on 19 October 2024)) were used for statistical analyses. The model selection was based on the Monte Carlo permutation test, allowing a maximum of three joinpoints to identify significant changes in trend direction. The software QGIS, version 3.10, was used to create maps. The *p*-value  <  0.05 was considered statistically significant. The results are presented as aggregate maps, graphs and tables, ensuring confidentiality of individual data.

According to national regulations, informed consent or ethics committee approval was not required, as the study was based on cases registered through routine epidemiological surveillance.

## 3. Results

### 3.1. Incidence and Mortality Rates of Acute and Chronic HBV in Vojvodina

**Figure 1:**
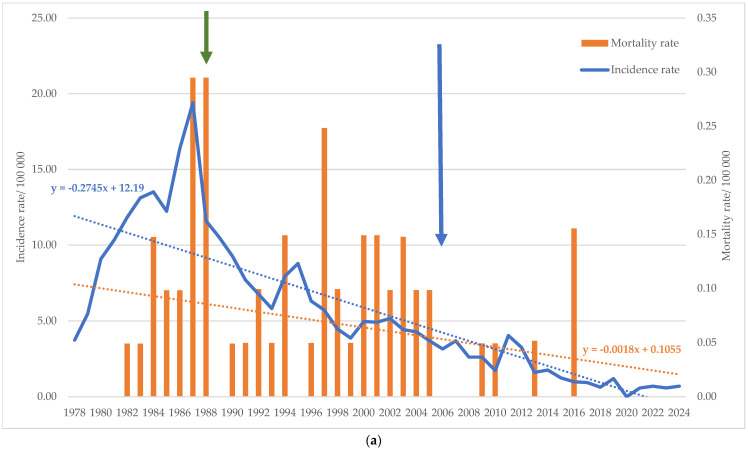

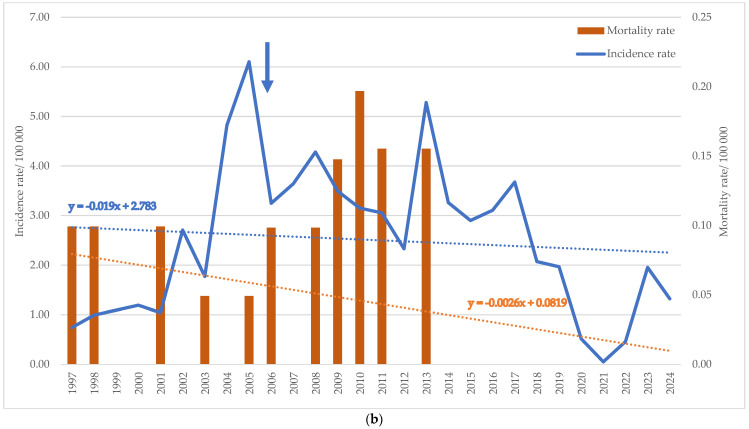
Incidence and mortality rates per 100,000 population of (**a**) acute HBV in the period 1978–2024 and (**b**) chronic HBV in the period 1997–2024, in Vojvodina, Serbia. The corresponding linear trendlines are also shown. The green arrow indicates the year of introduction of mandatory HBV immunization of newborns of HBsAg positive mothers and high-risk groups for HBV. The blue arrow represents the year of introduction of mandatory HBV immunization of newborns and infants and children at the age of 12.

Between 1978 and 2024, a total of 5328 cases of acute HBV were reported in Vojvodina, corresponding to the average incidence rate of 5.60 (95% CI: 4.27–6.96) per 100,000 population. Excluding the year 2020, when no cases of this disease were registered, the incidence rate of acute HBV ranged from 0.57/100,000 (2021 and 2023) to 19.41/100,000 (1987). In the pre-immunization period, the average incidence rate was 11.51 (95% CI: 8.16–14.86)/100,000. The average incidence rate decreased in the targeted and universal immunization period to 6.46 (95% CI: 5.28–7.63)/100,000 and 1.68 (95% CI: 1.10–2.26)/100,000, respectively. A total of 60 deaths related to acute HBV were registered (CFR = 1.13%). In the same period, the average mortality rate was 0.06 (95% CI: 0.04–0.09) per 100,000 population. The highest mortality rate was recorded in 1987 and 1988 (0.29/100,000). In the three periods (1978–1987; 1988–2005; 2006–2024), the average mortality rates of 0.07 (95% CI: 0.01–0.14)/100,000; 0.10 (95% CI: 0.07–0.14)/100,000 and 0.02 (95% CI: 0.00–0.04)/100,000 were reported, respectively. The incidence and mortality rate trends decreased over the observed period (Figure 1a).

In the period 1997–2024, a total of 1388 confirmed cases and 25 deaths (CFR = 1.80%) of chronic HBV were registered, with the average annual incidence rate of 2.51 (95% CI: 1.91–3.10) per 100,000 and the average mortality rate of 0.04 (95% CI: 0.02–0.07) per 100,000 population. The lowest incidence rate of 0.05/100,000 was recorded in 2021, and the highest of 6.10/100,000 in 2005. The mortality rate peaked in 2010 (0.20/100,000). The incidence and mortality rates of chronic HBV showed a decreasing trend (Figure 1b). The average incidence rates before universal immunization period (1997–2005) and in the period after it (2006–2024) were 2.27 (95% CI: 0.08–3.75) and 2.62 (95% CI: 1.97–3.27)/100,000, respectively, while average mortality rates were the same during both periods (0.04 (95% CI: 0.01–0.08; 95% CI: 0.04–0.16, respectively) per 100,000 population.

The results of the joinpoint regression analysis revealed a fluctuation in trends of acute HBV incidence rate in three joinpoints, connecting the four-line segment of the trend. The incidence rate of acute HBV significantly increased in the initial period (APC_1978–1983_ = 29.47%; p_trend_ < 0.05), then decreases in the following period, initially slightly (APC_1983–2017_ = −6.63%; p_trend_ < 0.05), then sharply (APC_2017–2020_ = −71.91%; p_trend_ < 0.05). In the subsequent period until 2024, a change in the trend direction was observed, with a significant sharp increase of 165.96% annually (p_trend_ < 0.05) (Figure S1a). The HBV mortality rate trend changed direction significantly in 1985 (APC_1978–1985_ = 137.92%; p_trend_ < 0.05 and APC_1985–2024_ = −12.29%; p_trend_ < 0.05) (Figure S2).

The joinpoint regression analysis indicated two segments of chronic HBV incidence rate trend with a significant change in the direction in 2008 from positive (APC_1997–2008_ = 19.67%; p_trend_ < 0.05) to negative (APC_2008–2024_ = −13.07%; p_trend_ < 0.05), showing a marked increase after 2006 (Figure S1b). The changes in the mortality rate trend of chronic HBV were not statistically significant (Figure S2).

**Figure 2:**
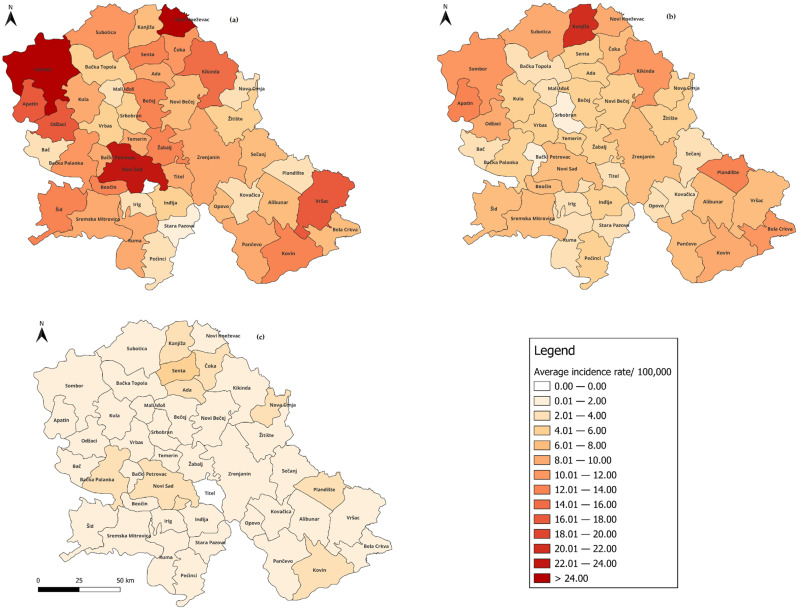
Average incidence rate of acute HBV by municipalities in Vojvodina, Serbia in the three periods: pre-immunization (**a**), targeted immunization (**b**) and universal immunization (**c**).

During the 47-year period, acute HBV was reported in all 45 municipalities in Vojvodina, with the highest average incidence rate of 10.13 (95% CI: 6.95–13.31)/100,000 registered in Sombor, followed by Apatin and Novi Kneževac (9.09 (95% CI: 5.55–12.64)/100,000 and 8.90 (95% CI: 4.56–13.24)/100,000, respectively)). The lowest average incidence rate was recorded in Stara Pazova (1.41 (95% CI: 0.86–1.95)/100,000). The average municipality-level incidence rates of acute HBV, divided into three periods, are shown in Figure 2. In the pre-immunization period, the average incidence rate ranged from 0.00 in municipality Sremski Karlovci to 24.99 (95% CI: 9.55–37.10) per 100,000 population in Novi Kneževac. In the targeted immunization period (1988–2005), the average incidence rate decreased in 32 out of 45 (71.11%) municipalities of Vojvodina by an average of 2.19 times. In the universal immunization period, the average incidence decreased in 42 (93.33%) municipalities by an average of 8.21 times compared to the pre-immunization period, and in 43 (95.56%) municipalities by an average of 4.68 times compared to the targeted immunization period (Figure 2). The average municipality-level mortality rate ranged from 0.00 to 0.89 (95% CI: −0.54–2.31)/100,000; 0.49 (95% CI: −0.26–1.24)/100,000 and 0.44 (95% CI: −0.48–1.35)/100,000 in three different periods, respectively (Figure S3).

**Figure 3:**
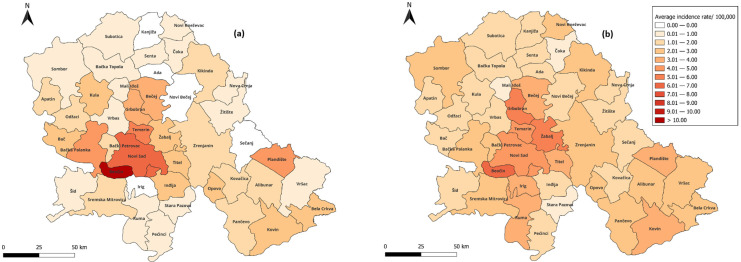
Average incidence rate of chronic HBV by municipalities in Vojvodina, Serbia in the two periods: targeted immunization (**a**) and universal immunization (**b**).

Similarly, chronic HBV was registered on the territory of all municipalities of Vojvodina. The average incidence rates varied from 0.28 (95% CI: 0.24–1.24) per 100,000 population in Stara Pazova to 7.52 (95% CI: 3.92–11.11) per 100,000 population in Beočin. In 33 (73.33%) municipalities, the average incidence rate increased 1.97 times on average, while in 12 (26.67%) municipalities, the incidence decreased by an average of 1.67 times between the targeted and universal immunization periods (Figure 3). In the period 1997–2024, the municipality-level mortality rate of chronic HBV peaked in Sremski Karlovci, followed by Opovo (0.47/100,000 (95% CI: −0.50–1.45); 0.32 (95% CI: −0.34–0.99)/100,000, respectively). The change in mortality rates between the two immunization periods occurred in 14 (31.11%) municipalities of Vojvodina, with half of them experiencing a decrease of 0.68 per 100,000 population on average, while the other half experienced an average increase of 0.14 per 100,000 population (Figure S4).

### 3.2. Gender and Age Specific Incidence Rates of Acute and Chronic HBV Cases in Vojvodina

**Figure 4:**
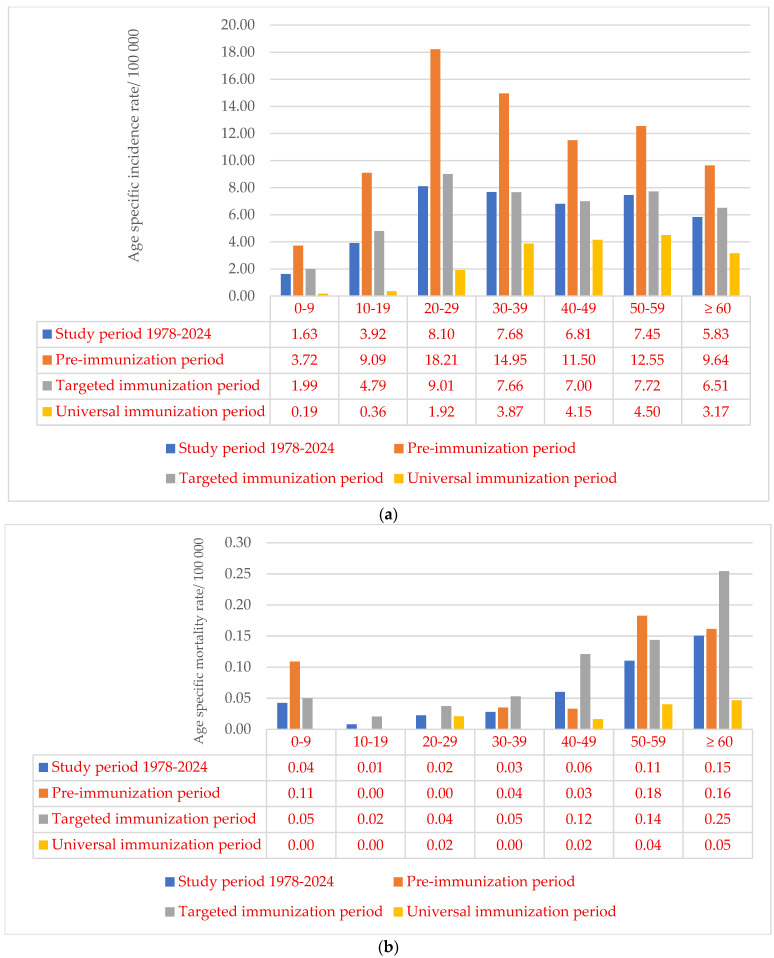
Age-specific incidence rate (**a**) and mortality rate (**b**) of acute HBV in Vojvodina, Serbia, in the period 1978–2024 and three periods (pre-immunization, targeted and universal immunization).

During the study period, males suffered from acute HBV slightly more often than females. Gender-specific incidence rate ratio (M:F) was 1.17 (6.08/100,000: 5.20/100,000). Acute HBV was registered in all age groups. The average specific incidence ranged from 1.63 (95% CI: 1.12–2.13)/100,000 in the 0–9 year age group, to 8.10 (95% CI: 5.98–10.21)/100,000 in those aged 20–29 years. Compared to the pre-immunization period, in the following periods, there was a decline in specific incidence rates in all age groups, with the largest decrease in the age groups 0–9 and 10–19 years (10.69 and 13.27 times, respectively, in the targeted immunization period and 19.95 and 25.16 times, respectively, in the universal immunization period) (Figure 4a). Death outcomes from acute HBV were registered in all age groups. The highest age-specific mortality rate of 0.15/100,000 was reported in the group of 60 and over years, followed by the 50–59 age group (0.11/100,000), with an evident decrease in rates in the universal immunization period in these age groups and in the age group up to 10 years (Figure 4b).

**Figure 5:**
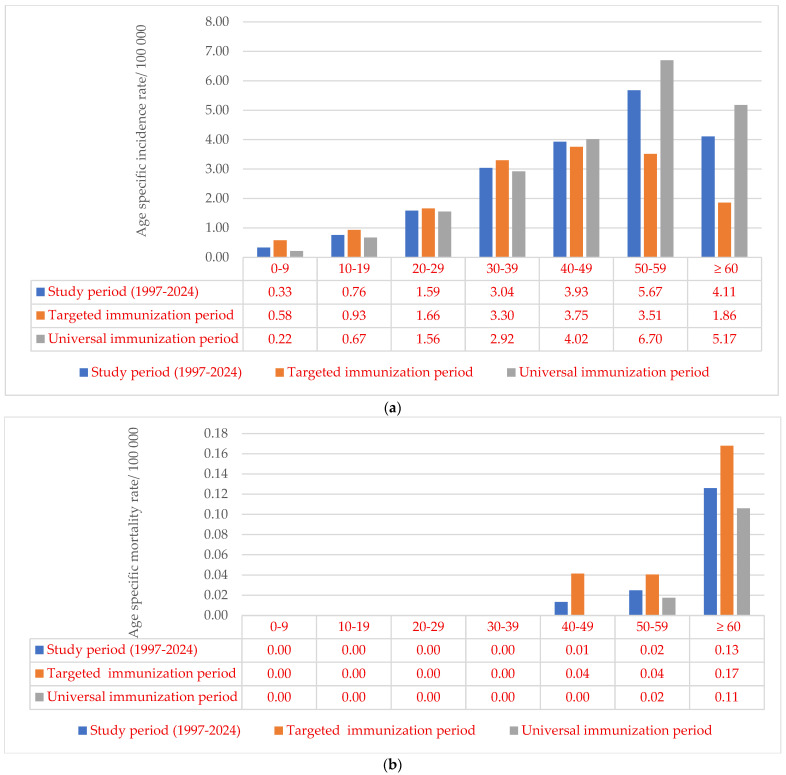
Age-specific incidence rate (**a**) and mortality rate (**b**) of chronic HBV in Vojvodina, Serbia, in the period 1997–2024 and two periods (targeted and universal immunization).

Similarly, chronic HBV was registered more often among males. The average gender-specific incidence for males was two times higher than the incidence recorded among females (M:F = 3.43/100,000: 1.64/100,000= 2.09:1). The highest age-specific incidence rate of chronic HBV was observed in the 50–59 age group (5.67/100,000), while the lowest of 0.33/100,000 was recorded in children under 10 years. During the universal immunization period (2006–2024), age specific incidence rates decreased by an average of 1.64 times in age groups up to 40 years, while they increased by an average of 1.15 times in older ages, compared to the previous targeted immunization period (Figure 5a). There were no reported deaths from chronic HBV under the age of 40. The highest specific mortality rate of 0.13 per 100,000 population was recorded in the age group of 60 and above. Between the study periods, twofold reduction in mortality rates across age groups was observed, where a death outcome was notified (Figure 5b).

According to the results of the joinpoint regression analysis, a statistically significant downward trend of the age-specific incidence rate of acute HBV was observed in all age groups, except for those of 30–39 and 40–49 years of age, during the study period. The statistically significant average annual percent change in the age-specific incidence rate varied from −12.05% in the 50–59 age group to −19.18% in the age group 10–19 years of age. In chronic HBV, the largest and only statistically significant trend decline of an average of 22.14% per year was recorded in the age group of 20 to 29 (Table 2, Figure S5).

### 3.3. HBV Vaccine Coverage Rates and the Effect of Vaccine Coverage on HBV Incidence Rate

**Figure 6:**
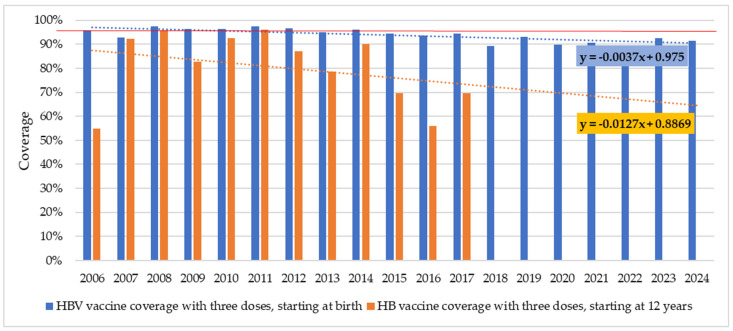
Hepatitis B vaccine coverage at birth in the period 2006–2024 and in the children at the age of 12 in the period 2006–2017, in Vojvodina, Serbia. The corresponding linear trendlines are shown. The red line depicts coverage of 95%.

In the period from 2006 to 2024, average HBV vaccine coverage in infants was 93.8% and varied between 88.6% (in 2022) and 97,3% (in 2008 and 2011), with a slightly descending trend. A coverage of over 95% was achieved only in seven years and was mainly registered at the beginning of this 19-year period. In children at the age of 12, in the period 2006–2017, HBV vaccine coverage ranged from 55.0% (2006) to 96.0% (2011) (average coverage 80.4%) with evident annual variations (Figure 6).

Results of the Poisson regression models revealed that the incidence rate of acute HBV in Vojvodina statistically significantly decreased by 2.20% for each increase in HBV vaccine coverage of 1% in infants in the previous year (IRR = 0.978; 95% CI: 0.975–0.981; *p* < 0.001). In the Poisson regression model, coefficient of HBV vaccine coverage was −0.0225 (95% CI: −0.0258 to −0.0192, *p* < 0.001) and constant was −8.462 (95% CI: −8.744 to −8.181). Goodness-of-fit statistics were as follows: Pseudo R^2^ = 0.150, deviance= 739.75, *p* < 0.001, AIC = 907.69. Likewise, each 1% increase in HBV vaccine coverage in the previous year led to a significant reduction in the incidence rate of chronic HBV by 1.80% (IRR = 0.982; 95% CI: 0.977–0.986; *p* < 0.001). Coefficient of HBV vaccine coverage was −0.0186 (95% CI: −0.0230 to −0.0141, *p* < 0.001) and constant was −8.940 (95% CI: −9.332 to −8.548). Goodness-of-fit statistics were as follows: Pseudo R^2^ = 0.0873, deviance= 475.45, *p* < 0.001, AIC = 632.033.

## 4. Discussion

In this paper, we report the indicators of HBV (incidence and mortality rate) retrieved from the national surveillance system over a 47-year period, analyzing acute and chronic HBV trends, vaccine coverage and the impact of HBV vaccination strategies. The results revealed that vaccination clearly has a long-term impact on reducing the incidence and mortality rate of HBV in Vojvodina. To the best of our knowledge, this study is the first extensive assessment of the epidemiology of HBV in the population of Vojvodina. Given that the epidemiology of HBV was assessed in a quarter of the national population, the results of the study are a good indicator of the current HBV status of the country and provide guidelines for future research.

During the study period, a downward trend in the incidence and mortality rates of acute and chronic HBV was observed in the territory of Vojvodina. This trend aligns with global HBV trends and is explained by the introduction of HBV vaccination and the interruption of mother-to-child transmission of HBV, and by the implementation of blood safety strategies in healthcare institutions, as well as other primary preventive measures [3,11,12]. A significant impact of vaccination on acute HBV in Vojvodina is also evidenced by the decrease in the average incidence and mortality rates observed after the introduction of immunization. Compared to the pre-immunization period, the average incidence rate of acute HBV decreased approximately twofold after the introduction of mandatory immunization of newborns of HBsAg-positive mothers and high-risk groups. Following the introduction of mandatory universal immunization of newborns, infants and children at the age of 12, the incidence further decreased nearly sevenfold. Similarly, the average mortality rate of acute HBV decreased approximately fivefold, compared to the pre-immunization period. While in Vojvodina, the incidence of acute HBV in post-vaccine period declined by ≈85%, in Italy, Poland and other European countries, the introduction of compulsory vaccination programs for newborns also led to a marked decrease in the incidence of acute HBV in subsequent years [21,22].

The impact of immunization on reducing the incidence of HBV in Vojvodina was also revealed by the results of the Poisson regression models. Therefore, we found that the IRR of acute and chronic HBV decreased statistically significantly by 2.20% and 1.80%, respectively, for each 1% increase in HBV vaccine coverage among infants in the previous year. Miglietta et al. demonstrated a more pronounced effect of vaccine coverage on acute HBV incidence at the EU/EEA level, showing a 10% decrease in acute HBV IRR for each 1% increase in vaccine coverage in the previous year. A statistically significant association between acute HBV reduction and increased vaccine coverage was observed in all EU/EEA countries. The IRR ranged from 5% in Estonia to 27% in the Czech Republic [20].

The recent surveillance data reported from EU/EEA showed a consistent decline in HBV incidence rates, which has been associated with the introduction of national HBV immunization programs in previous years [6,23]. The sharper decline in HBV incidence in 2020 in the EU/EEA countries was related to the reduced availability of HBV testing services and healthcare, with marked under-registration of all non-COVID infectious diseases, as well as reduced risk behaviors related to HBV, due to mitigating measures implemented during the COVID-19 pandemic [6,23,24]. The impact of COVID-19 mitigating measures was also evident in Serbia, with a sharp decline in acute and chronic HBV incidence during 2020–2022 (average incidence rate: 0.5/100,000 and 0.7/100,000, respectively) [10]. There were no reported cases of acute HBV in Vojvodina in 2020, while the average incidence rate of chronic HBV was seven and a half times lower in the period 2020–2022 compared to the average incidence registered in the preceding three years.

In addition to immunization, other primary prevention measures and the COVID-19 pandemic, fluctuations in the trend of acute HBV in Vojvodina were also influenced by improvements in epidemiological surveillance and greater awareness of the disease among reporting physicians. Increased availability of laboratory diagnostics also contributed to these fluctuations. Consequently, an increasing trend in acute HBV was observed during the initial period (1978–1983). In the United States of America, the slightly upward trend during 2013–2017 was influenced not only by improved surveillance, but also by the opioid crisis [25].

In our study, the results of the joinpoint regression analysis revealed a significant increase in the incidence trend of chronic HBV, rising by nearly 30% per year from the start of surveillance until 2008. Following this period, the trend showed a modest decline of approximately 7% per year. However, between 2017 and 2020, the incidence rate dropped sharply, resulting in a sharp annual trend decline of up to 72%. annually. Consistent with anticipated patterns, the period after 2020 was marked by a substantial upward shift in the trend, with an annual rise of 167%. The initial upward trend of chronic HBV observed in Vojvodina can be attributed to the previously unfavorable epidemiological situation and the absence of mandatory HBV vaccination in the past [9]. Nevertheless, the increase after 2006 may partly reflect improved diagnostic capacity, wider availability of laboratory testing, and updates to case definitions. The subsequent decline in the incidence trend was a result of HBV immunization, while the additional drop observed in the following years was likely a consequence of the COVID-19 pandemic.

The modest decline in chronic HBV incidence can be explained by a combination of several factors. These include the high incidence of acute HBV in the pre-vaccination era, vaccine non-response, and incomplete herd immunity due to gaps in vaccination coverage. Similarly, ongoing vertical transmission in areas with suboptimal vaccine uptake or high-risk behaviors among unvaccinated adults, such as unprotected sex or needle sharing, may have further mitigated the overall decline in chronic HBV incidence.

Similarly, in Poland, the trend of chronic HBV infection between 2005 and 2021 initially showed a slight increase, then a sharp rise, followed by a decline starting in 2017. The modest increase observed until 2013 was explained by the high incidence of acute HBV in the country during the 1980s and 1990s. The subsequent sharp rise in reported cases was attributable to significant legislative changes in Poland in 2014, introduced a wider case definition of HBV and made mandatory the reporting of positive HBV test results by laboratories [26,27].

Topographical disparities were observed in relation to the residence of patients with HBV in Vojvodina. Varying HBV incidence rates across municipalities may reflect differences in diagnostic availability and surveillance quality [9,10]. Local disparities in HBV incidence have also been noted in neighboring Croatia and elsewhere. In Croatia, significantly higher rates of HBV were reported in the Split-Dalmatia County compared to the Herzegovina-Neretva County. These differences are partly attributed to the geographical position of Split-Dalmatia County, which experiences higher population mobility, intense maritime traffic and tourism, which may facilitate risky behaviors [28]. In Brazil, a higher burden of HBV infection has been linked not only to variations in socioeconomic and healthcare factors, but also to the distribution of different HBV genotypes across regions [29]. The impact of the introduction of vaccination is also evident in the topographic distribution of acute HBV in Vojvodina. Accordingly, our findings revealed a widespread reduction in the incidence rate of acute HBV across nearly all municipalities during the last two periods (targeted and universal immunization periods), which highlights the critical role of vaccination in controlling HBV at the population level. By contrast, evidence of reduced chronic HBV incidence was found in fewer than one-third of the municipalities in Vojvodina. This may be explained by the fact that the effects of vaccination on chronic HBV become apparent only over a longer period, since mother-to-child transmission accounts for most chronic cases [4,9,30].

According to our results, the incidence of acute HBV was slightly higher in males compared to females, whereas the incidence of chronic HBV was twofold higher in males. This is consistent with reports from other countries, including those within the EU/EEA. The differences may be attributed to biological factors, health-seeking behaviors and the tendency of males to engage more frequently in risky behaviors [22,28,31,32].

Based on our findings, the highest incidence of acute HBV was recorded in the 20–59 age group, whereas chronic HBV was more frequently observed among persons over 50 years of age. In the EU/EEA countries, the highest rates of both acute and chronic HBV were seen among those aged between 25 and 55 [6,23,32]. The higher incidence of acute HBV in middle-aged groups can be explained by the higher levels of social and occupational activity in this age group, as well as by more frequent engagement in risk-related behaviors [22]. The higher frequency of chronic HBV among older age groups, who were not covered by universal immunization programs, may be partially attributed to iatrogenic infections. These were acquired through medical procedures, particularly those performed prior to the implementation of standardized infection control measures and routine HBV screening of blood products [33].

In the study period, age-specific incidence rates of acute HBV showed a significant decreasing trend in the age groups under 30 and over 49 years, with the largest decrease observed in those under 20 years of age. Similar to our results, a marked downward trend in the age-specific incidence rate of HBV was also observed in Croatia among adolescents and young adults (15–25 years of age), as a result of the early introduction of mandatory HBV vaccination in that country [34]. In China, between 2005 and 2021, a decreasing trend in acute HBV was also observed among the youngest age groups. However, the most significant decline occurred in the 15–39-year age group, which is explained by the early introduction of plasma-derived HBV vaccine in the country as early as 1985 [31]. In Poland, a significant decline in the incidence of acute HBV was observed across all age groups in the period 2005–2019. Consistent with our findings, this study also reported a notable reduction in the oldest age groups, suggesting that the improvement in the epidemiological situation is not solely the result of decreased incidence in cohorts covered by the mandatory universal vaccination program. Other preventive measures likely contributed as well [22].

In our study, chronic HBV showed only a statistically significant declining trend in the 20–29 age group. In contrast, a study from Poland reported no significant decline in chronic HBV cases only in the age group 65 years and older, suggesting that the effects of immunization programs are most evident in younger cohorts who have benefited from early vaccination coverage [26]. Differences between our findings and those from Poland may be attributed to the earlier and more comprehensive implementation of universal HBV vaccination in that country, as well as differences in historical exposure risks and the population age structure.

The HBV vaccine has been shown to be safe and effective with long-lasting protection for at least 30 years in more than 90% of vaccinated individuals [35]. Vaccination against HBV has been a cornerstone of HBV prevention for decades, but substantial barriers to achieving satisfactory vaccination coverage remain worldwide [36].

Between 2006 and 2024, vaccination coverage against HBV among infants in Vojvodina showed a slightly declining trend. Infant HBV vaccine coverage rates reached the desired levels (≥95%) in a few years and were above 90% in most of the years of the observed period. The anti-vaccine campaign and parents’ distrust and reluctance to vaccinate their children had some effect, but not sufficient to significantly influence the decline in rates [37]. Catch-up immunization at the age of 12 was completed in 2018, once the 2006 birth cohort reached the age of 12. The average coverage was 80.4% in the period 2006–2017. Although suboptimal, this coverage, together with the high HBV coverage in infancy, seems to be sufficient to influence the decline in incidence rates of acute HBV in age groups under 30. However, further serosurveys and targeted HBV vaccination efforts are needed to fill immunity gaps in the population in order to reduce HBV burden.

A decreasing trend in the incidence of acute and chronic HBV in Vojvodina was registered despite the downward trend in HBV mandatory vaccination coverage among newborns during 2006–2024 and among children at the age of 12 in the period 2006–2017. Since HBV in Vojvodina is most often registered at ages >20, the full effect of mandatory HBV vaccination on incidence rates could not be observed immediately [9].

According to the ECDC report, fewer than 40% of EU/EEA countries with a universal childhood vaccination program have reached the WHO target of 95% coverage, although most other countries are within 5% of the target. A more pronounced decline in vaccination coverage in these countries was observed during the COVID-19 pandemic [32,38].

In Serbia, perinatal prevention of HBV is part of routine prenatal care and includes mandatory screening of pregnant women for HBsAg in the third trimester and timely neonatal immune-prophylaxis. Although this policy is officially implemented, available data indicate significant regional variations in screening coverage (approximately 29.51–90.33% per district), suggesting that actual implementation remains suboptimal and that gaps in HBV prevention persist [15,39].

This study has several limitations. It relied on routine surveillance data collected over almost five decades, which may have been influenced by changes in reporting practices, variations in surveillance sensitivity, available diagnostic technologies and capacities, as well as case definitions, potentially leading to underestimation in some periods. Asymptomatic cases of HBV and HBsAg carriers often remain undiagnosed and consequently unreported in routine surveillance systems, which may have contributed to an underestimation of the disease burden. Incomplete laboratory confirmation in the earlier decades and evolving diagnostic assays may have led to inconsistencies in the application of case definitions over time. However, as all updates to case definitions have been conducted in accordance with international standards, this supports consistency in case classification and comparability of trends. Completeness of reporting was not formally assessed, nor were adjustments made for potential underreporting. Another limitation is the lack of individual-level linkage between vaccination records and disease outcomes. The ecological design does not allow assessment of vaccine effectiveness at the individual level, and the observed associations between vaccine coverage and incidence do not imply causation and should be interpreted with caution. Coverage estimates, especially for the 12-year cohort, showed variability and may have been affected by reporting inaccuracies. Furthermore, some estimates of annual percent change were based on small numbers and are statistically unstable. The absence of acute HBV cases in 2020 most likely reflects disruptions in health services during the COVID-19 pandemic rather than true elimination. In addition, due to the difficulties in conducting surveillance among migrants, this population was not included in our study. However, the results of this study are sufficient to characterize HBV incidence and mortality trends and to analyze the current epidemiology of HBV in Vojvodina. Despite limitations in reported surveillance data, it remains crucial to identify key populations to better guide the country-specific HBV elimination strategy.

By maintaining high infant HBV vaccination coverage, implementing universal maternal screening and timely neonatal prophylaxis, and addressing immunity gaps through targeted catch-up vaccination, Serbia could achieve the WHO HBsAg elimination target of <1% prevalence among children under five. Therefore, the results of this study are important when it comes to planning and improving prevention strategies in districts and municipalities of Vojvodina, where it is most needed, by raising awareness about the disease and educating the population on the importance of infant immunization and prevention measures, particularly among high-risk groups.

## 5. Conclusions

Over nearly five decades, HBV incidence and mortality in Vojvodina have declined substantially, particularly after the introduction of universal infant and adolescent immunization. The greatest impact was observed among children and adolescents, confirming the strong preventive effect of vaccination. High infant vaccine coverage was significantly associated with reductions in both acute and chronic HBV incidence, underscoring the importance of sustaining and enhancing programme performance. However, the persistent disease burden in older age groups, municipality-level incidence heterogeneity, and recent trend fluctuations highlight the need for ongoing surveillance, targeted interventions for susceptible cohorts, periodic review of booster vaccination policies among high-risk groups, and further studies—including serosurveys with continued monitoring of adult susceptibility, and molecular epidemiology—to better inform elimination strategies. Overall, the findings demonstrate the long-term public health benefits of HBV vaccination in Vojvodina, while also identifying to areas that require continued attention.

## Figures and Tables

**Table 1:** Study periods according to implementation HBV immunization in Serbia.

Number	Period Name	Years	Description
Period 1	Pre-immunization	1978–1987	Period before any immunization against HBV
Period 2	Targeted immunization	1988–2005	Period after the introduction of mandatory immunization of newborns of HBsAg positive mothers and high-risk groups for HBV
Period 3	Universal immunization	2006–2024	Period after the introduction of mandatory universal immunization of newborns, infants and children at the age of 12

**Table 2:** Average annual percent change in the age-specific incidence rate (per 100,000) of acute HBV in the period 1978–2024 and chronic HBV in the period 1997–2024 in Vojvodina, Serbia.

Acute HBV	**Age**	**AAPC** **(%)**	**Lower CI**	**Upper CI**	***p* Value**
	0–9	−16.62 *	−20.00	−11.91	**<0.000001**
	10–19	−19.18 *	−21.49	−15.60	**<0.000001**
	20–29	−13.98 *	−19.08	−9.94	**0.0024**
	30–39	−1.25	−7.68	3.78	0.69
	40–49	−0.61	−5.61	4.33	0.84
	50–59	−12.05 *	−17.86	−7.80	**0.01**
	≥60	−14.62 *	−18.40	−11.33	**<0.000001**
Chronic HBV	0–9	−0.17	−28.15	23.03	0.82
	10–19	−5.06	−19.48	13.41	0.52
	20–29	−22.14 *	−28.14	−17.52	**<0.000001**
	30–39	5.26	−3.97	14.67	0.22
	40–49	2.19	−6.22	9.94	0.42
	50–59	6.12	−2.03	14.03	0.13
	≥60	5.05	−2.64	10.02	0.14

* Indicates that annual percent change is significantly different from zero at the alpha = 0.05 level. Bold values indicate statistically significant results.

## Data Availability

The original contributions presented in this study are included in the article/supplementary material. Further inquiries can be directed to the corresponding author.

## Supplementary Materials

The following supporting information can be downloaded at: https://www.mdpi.com/article/10.3390/microorganisms13112504/s1, Figure S1: Joinpoint regression analysis plot of (a) acute HBV incidence rate trend in the period 1978–2024 and (b) chronic HBV incidence rate trend in the period 1997–2024 in Vojvodina, Serbia; Figure S2: Joinpoint regression analysis plot of (a) acute HBV mortality rate trend in the period 1978–2024 and (b) chronic HBV mortality rate trend in the period 1997–2024 in Vojvodina, Serbia; Figure S3: Average mortality rate of acute HBV by municipalities in Vojvodina, Serbia in the three periods: pre-immunization (a), targeted immunization (b) and universal immunization (c); Figure S4: Average mortality rate of chronic HBV by municipalities in Vojvodina, Serbia in the two periods: targeted immunization (a) and universal immunization (b); Figure S5: Average annual percent change in the age-specific incidence rate (per 100,000) of acute HBV in the period 1978–2024 and chronic HBV in the period 1997–2024 in Vojvodina, Serbia.
