# Supplementary material for: Long-Term Epidemiology of Hepatitis B and Impact of Vaccination in the Autonomous Province of Vojvodina, Serbia: A Population-Based Study
Source: Microorganisms. 2025 Oct 31;13(11):2504. doi: 10.3390/microorganisms13112504 (PMC12654862; doi:10.3390/microorganisms13112504)
Supplement: Supplementary file 1 [file microorganisms-13-02504-s001.zip › microorganisms-3928620-supplementary.pdf]

Supplementary Materials

The following supporting information can be downloaded at: www.mdpi.com/xxx/s1, **Figure S1.** Joinpoint regression analysis plot of a) acute HBV incidence rate trend in the period 1978-2024 and b) chronic HBV incidence rate trend in the period 1997-2024 in Vojvodina, Serbia; **Figure S2:** Joinpoint regression analysis plot of a) acute HBV mortality rate trend in the period 1978-2024 and b) chronic HBV mortality rate trend in the period 1997-2024 in Vojvodina, Serbia; **Figure S3**. Average mortality rate of acute HBV by municipalities in Vojvodina, Serbia in the three periods: pre-immunization (a), targeted immunization (b) and universal immunization (c); **Figure S4**. Average mortality rate of chronic HBV by municipalities in Vojvodina, Ser-bia in the two periods: targeted immunization (a) and universal immunization (b); **Figure S5.** Average annual percent change in the age-specific incidence rate (per 100 000) of acute HBV in the period 1978-2024 and chronic HBV in the period 1997-2024 in Vojvodina, Serbia.

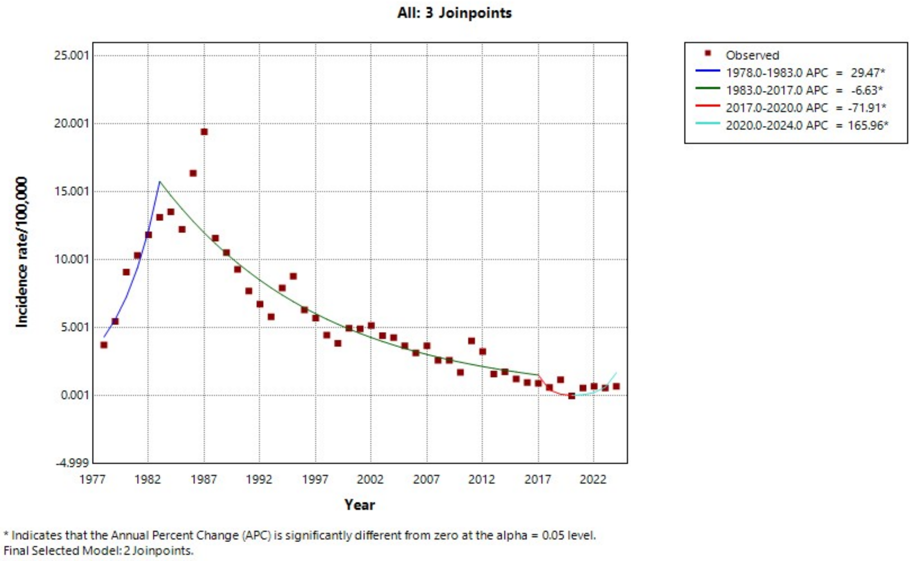

(a)

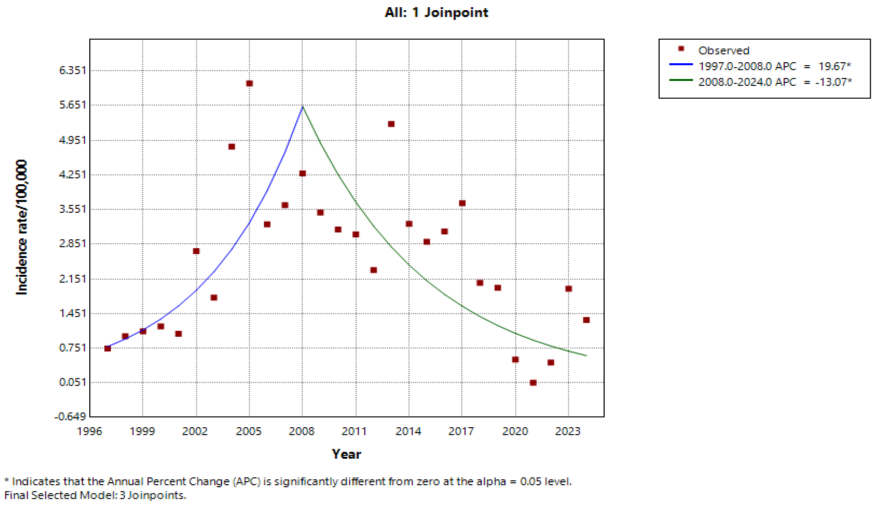

(b)

Figure S1. Joinpoint regression analysis plot of a) acute HBV incidence rate trend in the period 1978-2024 and b) chronic HBV incidence rate trend in the period 1997-2024 in Vojvodina, Serbia

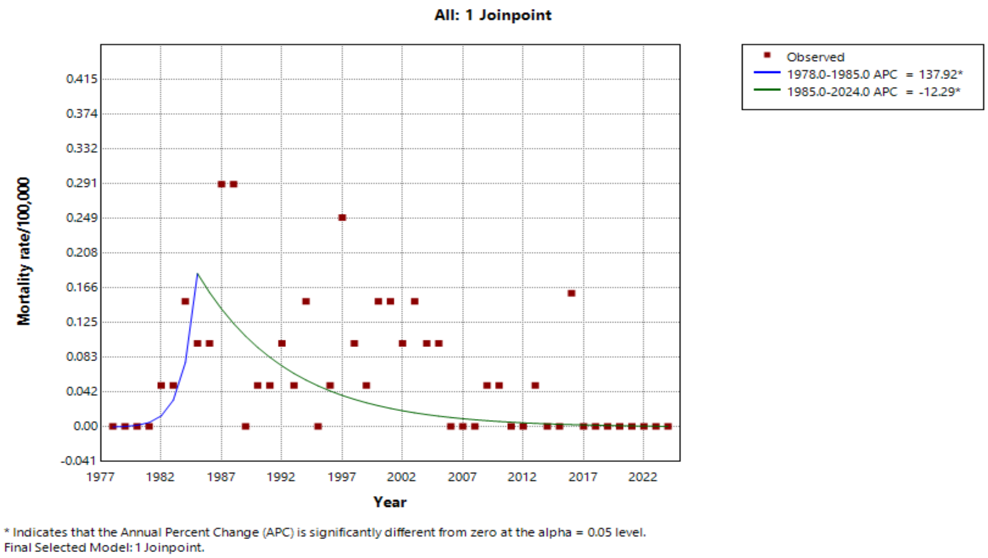

(a)

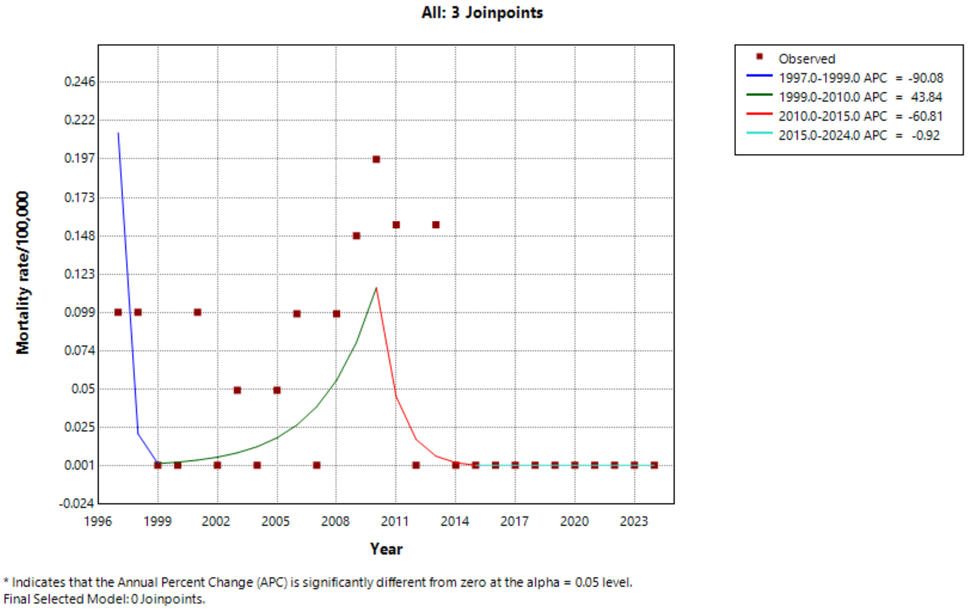

(b)

Figure S2. Joinpoint regression analysis plot of (a) acute HBV mortality rate trend in the period 1978-2024 and (b) chronic HBV mortality rate trend in the period 1997-2024 in Vojvodina, Serbia

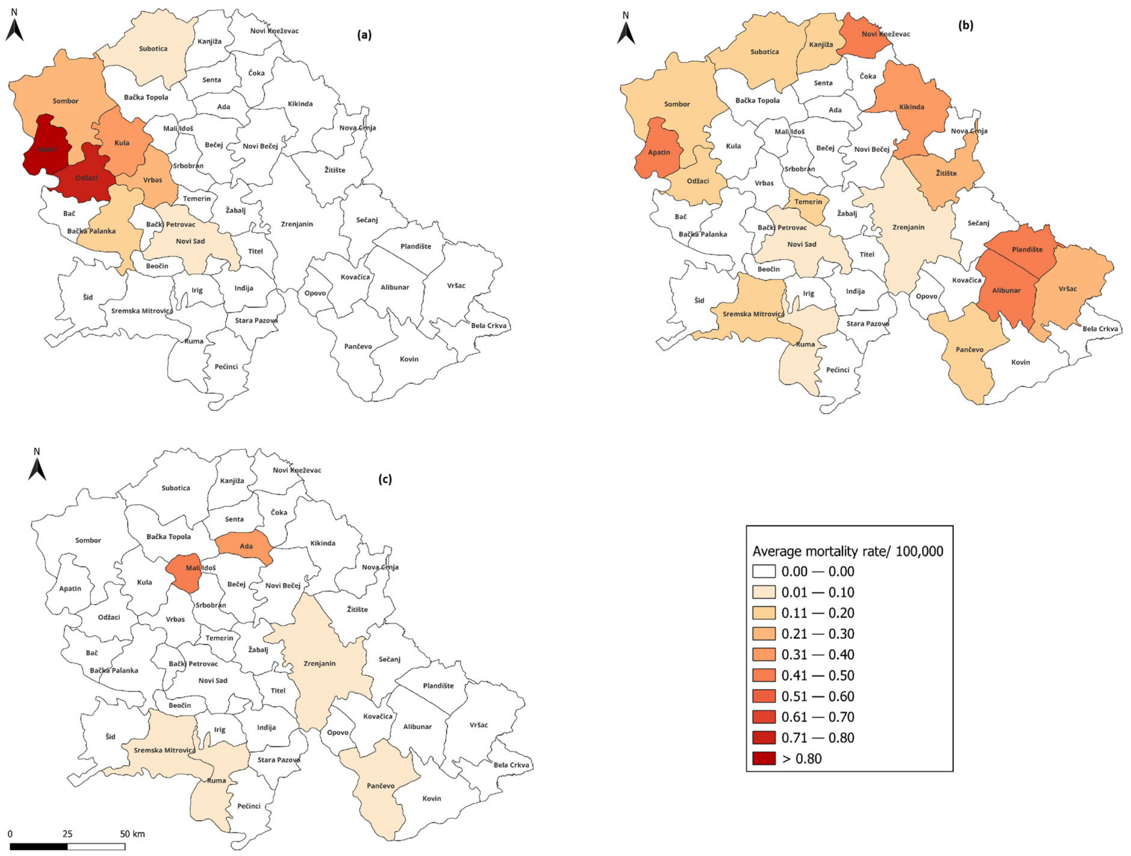

Figure S3. Average mortality rate of acute HBV by municipalities in Vojvodina, Serbia in the three periods: pre-immunization (a), targeted immunization (b) and universal immunization (c)

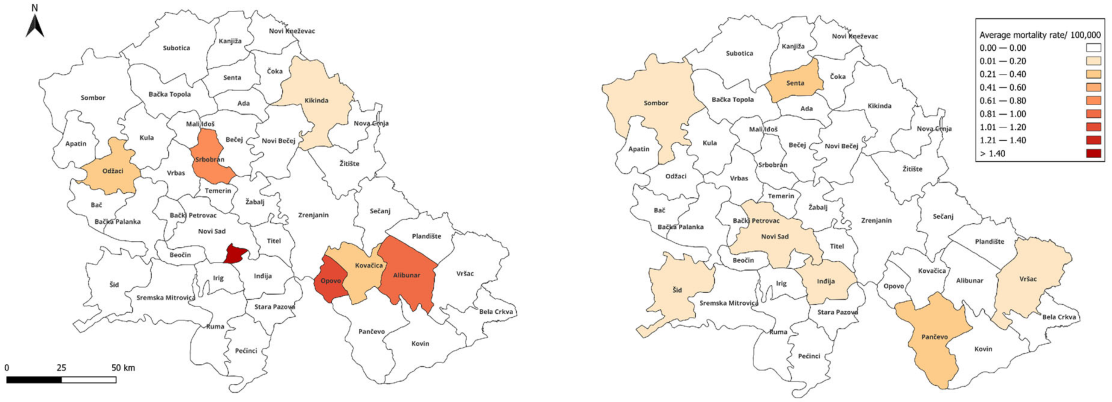

(a) (b)

Figure S4. Average mortality rate of chronic HBV by municipalities in Vojvodina, Serbia in the two periods: targeted immunization (a) and universal immunization (b)

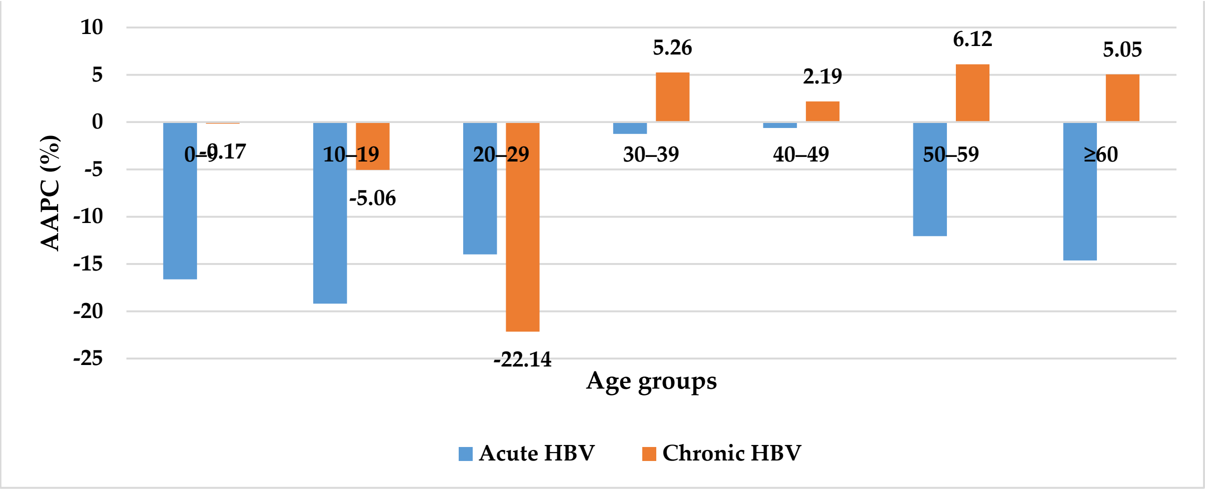

Figure S5. Average annual percent change in the age-specific incidence rate (per 100 000) of acute HBV in the period 1978-2024 and chronic HBV in the period 1997-2024 in Vojvodina, Serbia
